# The Relationship of Vitamin A and Neonatal Respiratory Diseases: A Meta‐Analysis

**DOI:** 10.1111/crj.70030

**Published:** 2024-10-21

**Authors:** Yuanyuan Li, Ruoyu Zhang, Zhongliang Li, Qingfeng Zhai

**Affiliations:** ^1^ Department of Neonatology Weifang Maternal and Child Health Hospital Weifang China; ^2^ School of Public Health Hebei Medical University Shijiazhuang China; ^3^ School of Public Health Shandong Second Medical University Weifang China

**Keywords:** meta‐analysis, neonatal respiratory diseases, vitamin

## Abstract

This study systematically analyzes the relationship of vitamin A on the neonatal respiratory diseases. An extensive literature search for relevant studies was conducted on PubMed, Web of Science, and so on. After screening in strict accordance with the inclusion and exclusion criteria, 12 articles on vitamin A deficiency and 12 articles on vitamin A supplementation were included. Stata 17.0 software was used to perform meta‐analysis, heterogeneity test, and sensitivity analysis, and the corresponding mathematical model was used to merge the data. The meta‐analysis results of the relationship between vitamin A deficiency and neonatal respiratory diseases indicated that compared with the neonates with normal vitamin A, the neonates with vitamin A deficiency had adverse health outcomes of neonatal respiratory diseases (OR = 4.86, 95% CI: 2.68–8.84), of which neonatal respiratory distress syndrome (NRDS) (OR = 4.10, 95% CI: 2.32–7.23) and neonatal pneumonia (OR = 3.22, 95% CI: 2.18–4.77) were analyzed by subgroup analysis. The meta‐analysis of the relationship between vitamin A supplementation therapy and neonatal respiratory diseases showed that vitamin A supplementation was an effective therapeutic measure for neonatal respiratory diseases (RR = 1.06, 95% CI: 1.04–1.07): NRDS (RR = 1.03, 95% CI: 1.02–1.05) and NBPD (RR = 1.08, 95% CI: 1.01–1.15). The funnel chart method results show that there was publication bias in studies on vitamin A deficiency induced to and vitamin A supplementation therapy for neonatal respiratory diseases. The sensitivity analysis results showed that excluding some special article had some effect on the final pooled effect. But generally speaking, the result of meta‐analysis was stable. There is a statistical correlation of vitamin A on the neonatal respiratory diseases from two aspects of etiological exploration and effect evaluation of treatment.

## Graphical abstract

This study systematically analyzes the relationship of vitamin A on the neonatal respiratory diseases from two aspects of etiological exploration and effect evaluation of treatment. In conclusion, for neonatal respiratory related diseases, low serum vitamin A level was a risk factor, and vitamin A supplementation was an effective treatment.

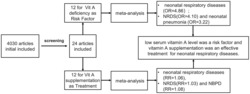

## 1 Introduction

Neonatal health is an important topic for human development. Exploring the etiology and treatment of neonatal diseases, especially respiratory diseases, has always been the focus of research in the field of health [1, 2]. Vitamin A is a group of bioactive β‐violanone derivatives, including retinol, retinaldehyde, retinoic acid, and retinoid ester. Vitamin A is responsible for the growth and differentiation and integrity of epithelial cells and also plays an important role in the development of the lungs and retina.

Since the second half of the 20th century, more clinical analysis and studies had found that preterm birth often leads to low serum vitamin A content and preterm infants are often accompanied by respiratory diseases, including neonatal asphyxia, neonatal pneumonia, neonatal bronchitis, and neonatal respiratory tract infection [3]. In recent years, studies have shown that retinoic acid and retinol can also promote lung development [4], which further confirms that vitamin A may affect respiratory system related diseases in newborns. But Zhang et al. [5] concluded that vitamin A supplementation according to WHO recommendations has little value for children aged 0–11 years in the prevention of or recovery from acute respiratory infections, and it may cause a temporary immune dysregulation and lead to increased risks of infectious diseases when provided in excess of the WHO's recommended dose.

Briefly, there were many studies on the relationship of vitamin A and neonatal respiratory diseases, but the conclusions were still inconsistent. The purpose of this study was to explore the effect of vitamin A on neonatal respiratory diseases by searching and systematically evaluating the published literature about neonatal respiratory diseases caused by vitamin A deficiency or treated with vitamin A supplementation.

## 2 Methods

### 2.1 Data Source, Search Strategy, and Selection Criteria

An extensive literature search for relevant studies was conducted on PubMed, Web of Science, Excerpta Medica Database, the Chinese National Knowledge Infrastructure, and Chinese Biomedical Literature Database from January 1, 1990, to December 11, 2022. Search terms included “newborn,” “infant,” “premature,” “preterm,” “Vitamin A,” “retinol,” “respiratory disease,” “respiratory disorder,” “respiratory distress syndrome,” “bronchopulmonary dysplasia,” “lung disease,” and “Pneumonia.” No language restrictions were implemented. Moreover, other relevant literature was manually retrieved and traced.

Literature inclusion criteria were as follows: original data literature; at least one control group, which was comparable in influencing factors with exposure group; observation of at least one respiratory related adverse health outcomes; results were expressed as count data; and there were a clear total number of exposure group and control group and the number of adverse outcomes.

Literature exclusion criteria were as follows: those who did not meet the above inclusion criteria; repeated literature; review and critical articles; lack of neonatal‐related diseases in the study; the design of the study is unreasonable and lacks the control group; animal experimental research; full text is not available or the article contains the same data as another article.

### 2.2 Data Collection and Quality Assessment

Microsoft Office 365 Excel was used to collect and collate the following data, including author, publication year, study time, study design, the prevalence of neonatal respiratory diseases (such as neonatal pneumonia, neonatal respiratory distress syndrome, neonatal respiratory tract infection, and neonatal bronchopulmonary dysplasia), sample size, infant sex, the serum vitamin A levels, and the therapeutic effect of vitamin A. The Newcastle–Ottawa Scale (NOS) was used to evaluate the literature quality of included cohort studies and case–control studies, and the Jadad scale was used to evaluate the randomized controlled trial.

According to the serum levels of vitamins A and E, the neonates were divided into vitamin A deficiency group (Vit A < 0.35 μmol/L), vitamin A normal group (Vit A ≥ 0.35 μmol/L), Vit E deficiency group (Vit E < 5 mg/L), and vitamin E normal group (Vit E ≥ 5 μmol/L).

### 2.3 Statistical Analysis Methods

The meta‐analysis was carried out with Stata 17.0 software. Odds ratio (OR) and their 95% CI were expressed as the effect size for case–control studies, and relative risk (RR) and their 95% CI were expressed as effect sizes for cohort or randomized controlled studies. The heterogeneity of the included literature was tested and quantified by *I* ^2^ value. If there was no significant heterogeneity (*I* ^2^ ≤ 50%), the fixed‐effects model was used for the combined analysis; otherwise, random‐effects model was used (*I* ^2^ ≥ 50%). The publication bias was evaluated according to the asymmetry of funnel plot. To assess the impact of each individual study on the overall estimates for the rest of the studies, the leave‐one‐out sensitivity analysis was repeated by deleting one study at a time to confirm that the findings were not affected by any individual study. The inspection level was 0.05.

### 2.4 Basic Information of the Included Literature

**FIGURE 1:**
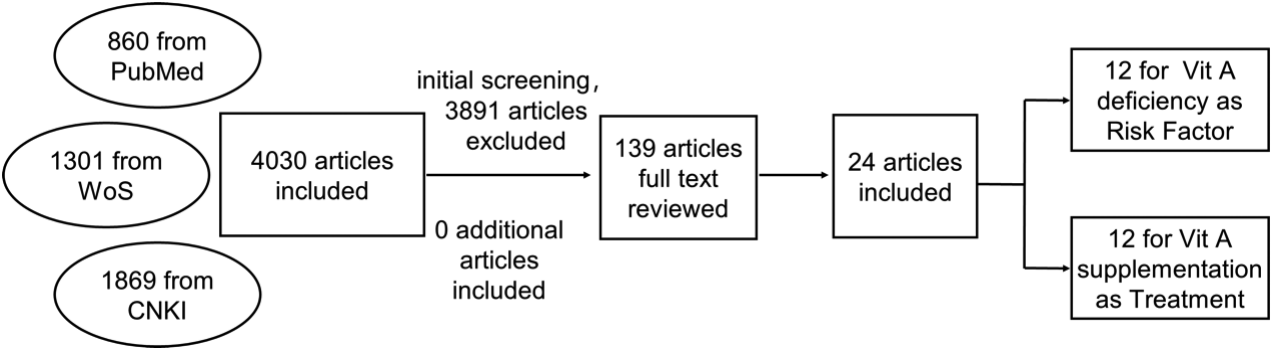
Article citation process.

A total of 4030 articles on the relationship between vitamin A and neonatal respiratory diseases were retrieved. After initial screening, 139 valuable articles were obtained. The full text of the 139 articles was read and screened in strict accordance with the inclusion and exclusion criteria. Finally, 24 articles were included in this study, including 12 articles on vitamin A deficiency as a risk factor and 12 articles on vitamin A supplementation as a means of treatment. The screening process is as shown in Figure 1. The basic characteristics of the included literature are shown in Tables 1 and 2.

**TABLE 1:** Basic information of vitamin A deficiency as a risk factor groups.

Study	Country	Research year	Disease	Number	NOS/Jadad
Jiang (2017) [6]	China	2013–2015	NRDS	82	7
Gu et al. (2015) [7]	China	2013	Pneumonia	169	6
Dai et al. (2018) [8]	China	2016–2017	Pneumonia	100	6
Wang and Xing (2017) [9]	China	2014–2016	NRDS	90	7
Yang et al. (2017) [10]	China	2014–2016	NRDS	156	6
Jiang and Peng (2016) [11]	China	2014–2015	Pneumonia	88	7
Jiang and Peng (2016) [11]	China	2014–2015	RTI	99	7
Cheng and Bao (2015) [12]	China	2013–2014	NRDS	166	6
Verma et al. (1996) [13]	America	1996	BPD	22	6
Li and Zhang (2021) [14]	China	2019–2020	Pneumonia	86	6
Chen (2020) [15]	China	2016–2018	Pneumonia	1270	6
Tao et al. (2021) [16]	China	2016–2019	NRDS	208	7
Zhang et al. (2022) [17]	China	2018–2019	NRDS	310	8

Abbreviations: BPD, bronchopulmonary dysplasia; NRDS, neonatal respiratory distress syndrome; RTI, respiratory tract infection.

**TABLE 2:** Basic information of vitamin A supplement as a means of treatment groups.

Study	Country	Research year	Disease	Number	NOS/Jadad
Donnen et al. (2007) [18]	Senegal	2006–2007	NRDS	1214	5
Si et al. (1997) [19]	Vietnam	1997	Pneumonia	592	4
Garofoli et al. (2018) [20]	Italy	2014–2016	BPD	41	5
Basu et al. (2019) [21]	India	2017–2019	BPD	196	6
Abdeljaber et al. (1991) [22]	Indonesia	1982–1983	Cough	428	4
Pearson et al. (1992) [23]	Carolina	1992	NRDS	49	4
Grubesic and Selwyn (2003) [24]	Nepal	2000	RTI	641	4
Stansfied et al. (1993) [25]	Canada	1993	NRDS	15 344	5
Stansfied et al. (1993) [25]	Canada	1993	Cough	15 344	5
Stansfied et al. (1993) [25]	Canada	1993	Rhinitis	15 344	5
Stansfied et al. (1993) [25]	Canada	1993	Flu	15 344	5
Pearson et al. (1992) [23]	Carolina	1992	BPD	49	4
Kiatchoosakun et al. (2014) [26]	Thailand	1995–1997	BPD	80	5
Chang et al. (2006) [27]	Australia	2001–2002	RTI	225	6
Li et al. (2021) [28]	China	2017–2020	NRDS	96	7
Rakshasbhuvankar et al. (2021) [29]	Australia	2016–2019	BPD	188	7

Abbreviations: BPD, bronchopulmonary dysplasia; NRDS, neonatal respiratory distress syndrome; RTI, respiratory tract infection.

## 3 Results

### 3.1 Meta‐Analysis of the Relationship Between Vitamin A Deficiency and Neonatal Respiratory Diseases

**FIGURE 2:**
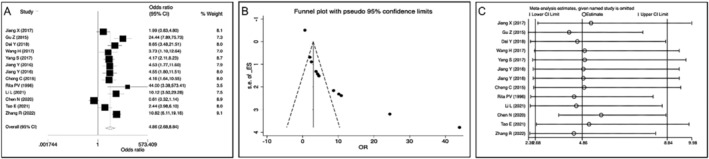
Meta‐analysis of the relationship between vitamin A deficiency and neonatal respiratory diseases. (A) Forest plot. (B) Funnel plot. (C) Sensitivity analysis.

In the study of vitamin A deficiency and neonatal respiratory diseases, the data of 12 included articles were entered into Stata 17 software, and the results of meta‐analysis were obtained. The epidemiological methods used in the study of vitamin A deficiency and neonatal respiratory diseases were all case–control studies. The study included 13 studies in 12 literatures, with 2846 participants and *I* ^2^ = 68.31% ≥ 50%. The random‐effects model was used to analyze the pooled research data caused by vitamin A deficiency to reduce bias: pooled OR = 4.86, 95% CI: 2.68–8.84, Z = 5.19, *p* < 0.01, indicating that vitamin A deficiency is a risk factor for neonatal respiratory diseases (Figure 2A).

**FIGURE 3:**
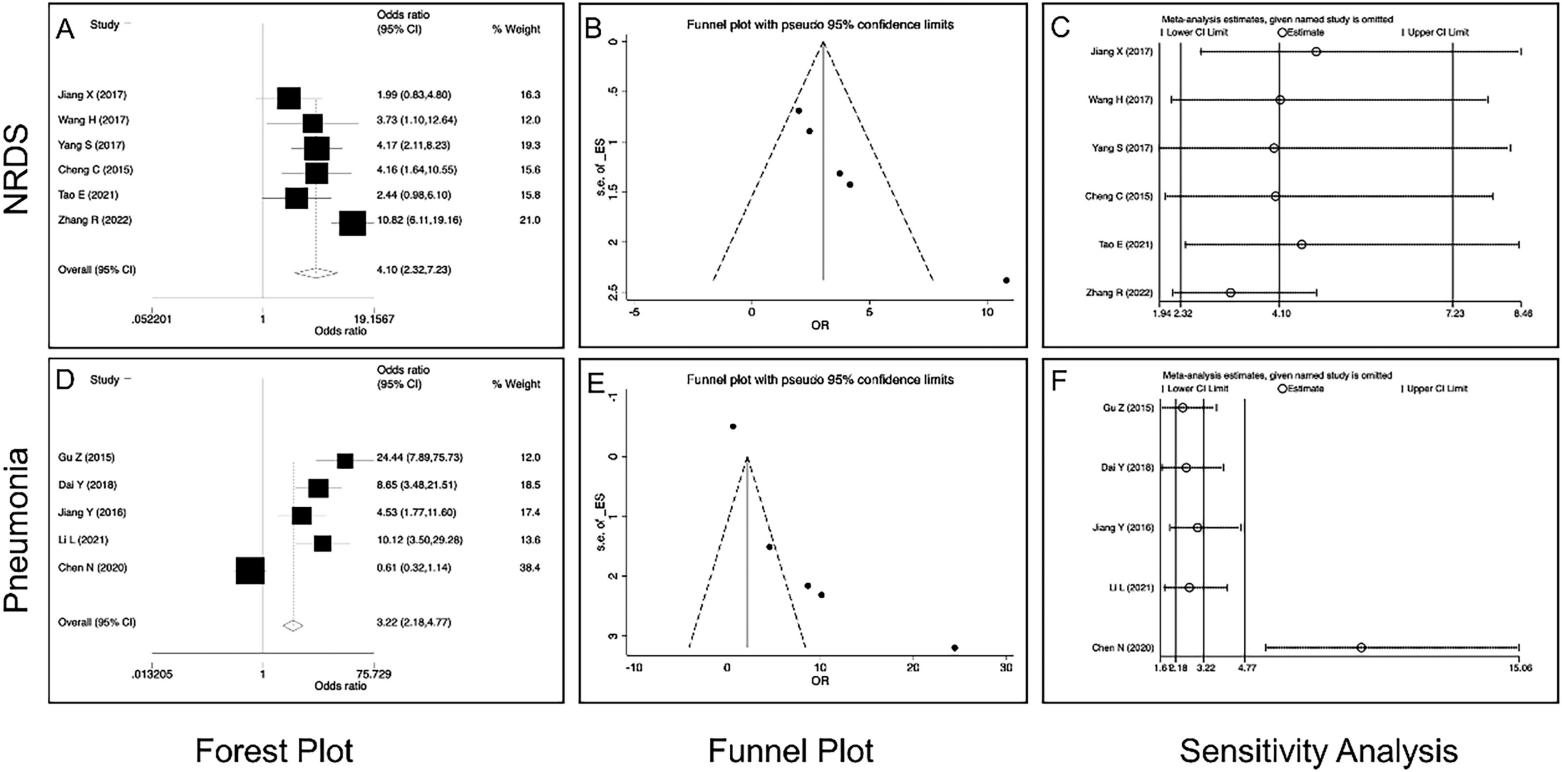
Subgroup analysis of the relationship between vitamin A deficiency and neonatal respiratory distress syndrome (NRDS) or neonatal pneumonia. (A) Forest plot of NRDS. (B) Funnel plot of NRDS. (C) Sensitivity analysis of NRDS. (D) Forest plot of neonatal pneumonia. (E) Funnel plot of neonatal pneumonia. (F) Sensitivity analysis of neonatal pneumonia.

In the study of vitamin A deficiency and neonatal respiratory diseases, neonatal respiratory distress syndrome (NRDS) and neonatal pneumonia were the most studied, which were analyzed by subgroup analysis: NRDS (6 literatures, 6 studies, *n* = 1012), *I* ^2^ = 14.11% < 50%; pooled OR = 4.10, 95% CI: 2.32–7.23, Z = 9.44, *p* < 0.01, indicating that vitamin A deficiency was a risk factor for NRDS (Figure 3A), and neonatal pneumonia (5 literatures, 5 studies, *n* = 1713), *I* ^2^ = 48.64% < 50%; pooled OR = 3.22, 95% CI: 2.18–4.77, Z = 5.85, *p* < 0.01, indicating that vitamin A deficiency was a risk factor for neonatal pneumonia (Figure 3D).

### 3.2 Publication Bias and Sensitivity Analysis of the Relationship Between Vitamin A Deficiency and Neonatal Respiratory Diseases

The potential bias in meta‐analysis was evaluated, including the identification and detection of publication bias. In this study, the funnel plot method was used to evaluate the possible publication bias. The included articles suffered from publication bias on vitamin A deficiency and neonatal respiratory diseases (Figure 2B).

Sensitivity analysis was performed to assess the effect of every study on the value of the pooled risk estimate by sequentially excluding one study in one turn. The results of sensitivity analysis showed that excluding any one article had no effect on the pooled risk estimate, indicating that the result of meta‐analysis was stable (Figure 2C).

In the subgroup analysis of NRDS and neonatal pneumonia, the results of funnel chart analysis showed that publication bias was existed in both NRDS and neonatal pneumonia (Figure 3B,E). The sensitivity analysis results showed that excluding any one article had no effect on the final pooled OR value in NRDS but had some influence in neonatal pneumonia, especially Chen's research [15] (Figure 3C,F). It may be mainly attributed to relatively less neonatal pneumonia literatures included in the study and the large number of studies in Chen [15].

### 3.3 Meta‐Analysis of the Relationship Between Vitamin A Supplementation Therapy and Neonatal Respiratory Diseases

**FIGURE 4:**
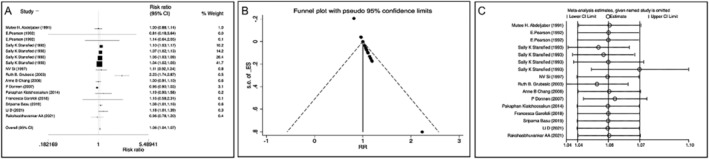
Meta‐analysis of the relationship between vitamin A supplementation therapy and neonatal respiratory diseases. (A) Forest plot. (B) Funnel plot. (C) Sensitivity analysis.

In the study of vitamin A supplementation therapy and neonatal respiratory diseases, the data of 12 included articles were entered into Stata software, and the results of meta‐analysis are as follows. All the literature included in this study used cohort study and randomized controlled study. The study included 16 studies in 12 literatures, with 19 094 participants and *I* ^2^ = 58.05% ≥ 50%. The random‐effects model was used to analyze the pooled research data caused by vitamin A supplementation therapy to reduce bias: pooled RR = 1.06, 95% CI: 1.04–1.07, Z = 7.77, *p* < 0.01, indicating that vitamin A supplementation was an effective therapeutic measure for neonatal respiratory diseases (Figure 4A).

**FIGURE 5:**
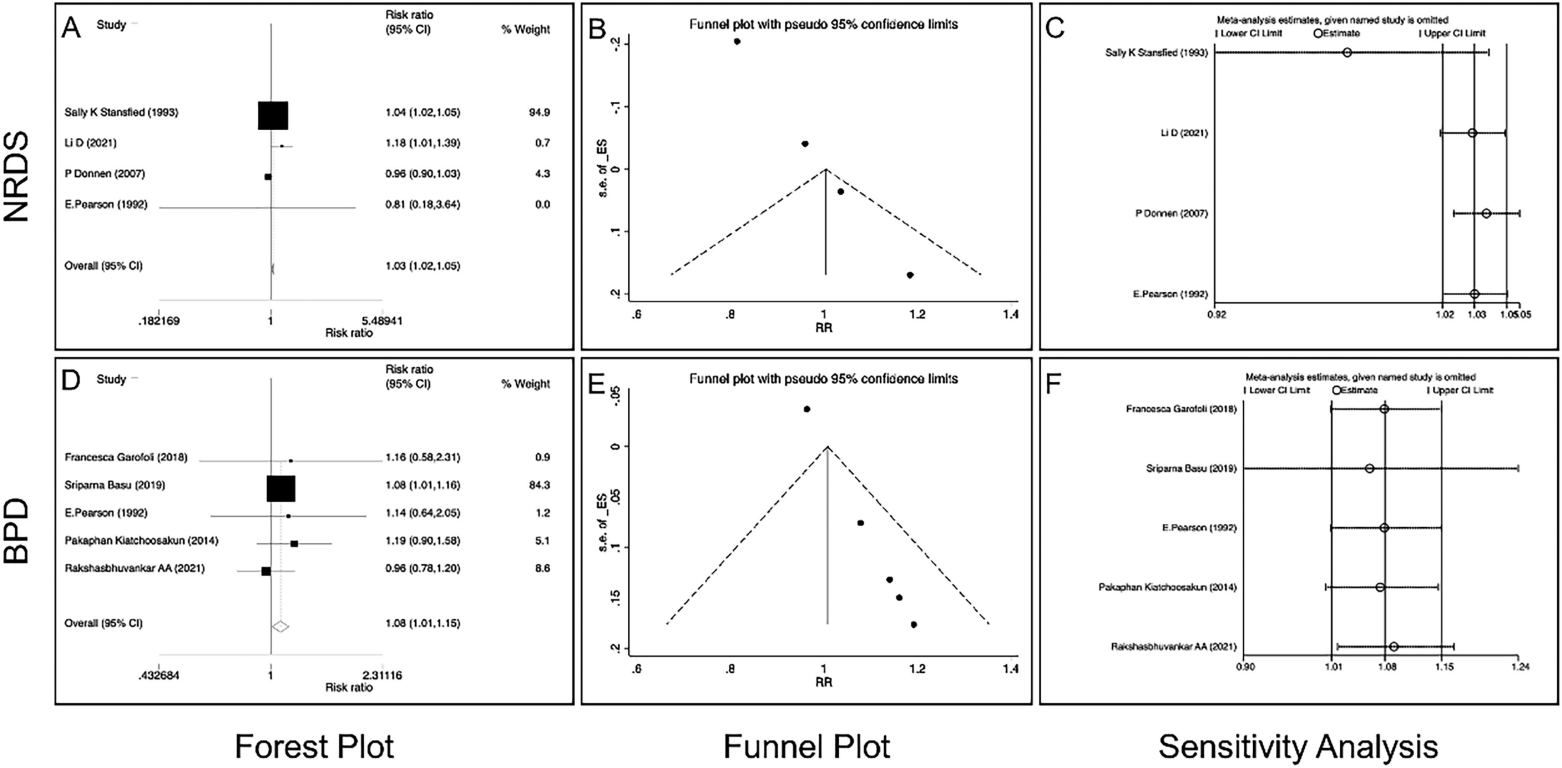
Subgroup analysis of the relationship between vitamin A supplementation therapy and neonatal respiratory distress syndrome (NRDS) or neonatal bronchopulmonary dysplasia (NBPD). (A) Forest plot of NRDS. (B) Funnel plot of NRDS. (C) Sensitivity analysis of NRDS. (D) Forest plot of NBPD. (E) Funnel plot of NBPD. (F) Sensitivity analysis of NBPD.

In the study of vitamin A supplementation therapy and neonatal respiratory diseases, NRDS and neonatal bronchopulmonary dysplasia (NBPD) were the most studied, which were analyzed by subgroup analysis: NRDS (4 literatures, 4 studies, *n* = 16 703), *I* ^2^ = 7.80% < 50%; pooled RR = 1.03, 95% CI: 1.02–1.05, Z = 4.79, *p* < 0.01, indicating that vitamin A supplementation was an effective therapeutic measure for NRDS (Figure 5A), and NBPD (5 literatures, 5 studies, *n* = 554), *I* ^2^ = 1.59% < 50%; pooled RR = 1.08, 95% CI: 1.01–1.15, Z = 2.24, *p* < 0.05, indicating that vitamin A supplementation was an effective therapeutic measure for NBPD (Figure 5D).

### 3.4 Publication Bias and Sensitivity Analysis of the Relationship Between Vitamin A Supplementation Therapy and Neonatal Respiratory Diseases

The evaluation results of the funnel chart method show that there was publication bias in studies on vitamin A supplementation therapy for neonatal respiratory diseases (Figure 4B). But the sensitivity analysis results showed that excluding any one article had no effect on the value of the pooled risk estimate, indicating that the result of meta‐analysis on the effect of vitamin A supplementation therapy on neonatal respiratory diseases was stable (Figure 4C).

In the subgroup analysis of vitamin A supplementation therapy for NRDS and NBPD, the funnel chart analysis results showed that there were publication biases in both two. The sensitivity analysis results showed that excluding any one article had no effect on the final pooled effect in NBPD but had some influence in NRDS, especially Stansfied et al.'s research [25]. The main reason may be that there were relatively few studies included in the subgroup analysis and the large number of studies in Stansfied et al. [25]. After excluding this study, vitamin A supplementation was still an effective treatment of NRDS and BPD (RR > 1) (Figure 5C,F).

## 4 Discussion

In recent years, there are many epidemiological studies on the effects of vitamin A on neonatal respiratory diseases; however, there is still no definitive conclusion yet. In this study, we collected the published literature on neonatal respiratory diseases induced by vitamin A deficiency and treated with vitamin A supplementation. And then the meta‐analysis was used to comprehensively analyze the relationship between vitamin A and neonatal respiratory diseases from two aspects of etiological exploration and effect evaluation of treatment.

In this study, meta‐analysis of vitamin A deficiency and neonatal respiratory diseases showed that low serum vitamin A level was a risk factor for neonatal respiratory related diseases, and subgroup analysis confirmed that vitamin A deficiency was a significant risk factor for NRDS and neonatal pneumonia. Meanwhile, meta‐analysis of vitamin A supplementation and neonatal respiratory diseases showed that vitamin A supplementation was an effective treatment for neonatal respiratory diseases. The results of subgroup analysis also showed that although there was publication bias, vitamin A supplementation was still confirmed as a protective factor for NRDS and NBPD.

Similar to the results of this study, West et al. have shown that there is a certain association between vitamin A and the mortality of preterm newborns, especially in very low birth weight newborns [30]. Ahmad et al. have confirmed that neonatal serum vitamin A can effectively enhance the immunity of pregnant women and newborns and artificial supplementation of vitamin A can reduce neonatal respiratory tract infection and other related diseases [31, 32]. Comprehensive analysis combined with this study, neonatal respiratory diseases as the main cause of neonatal death, can be improved by vitamin A supplementation and then reduce neonatal death.

Meta‐analysis has been recognized as an effective method to answer a wide variety of clinical questions by summarizing and reviewing previously published quantitative research. With the concept of evidence‐based medicine and the research of evidence as the decision‐making basis, the results are more real and accurate than the traditional single independent research results. Meanwhile, because the data collected by meta‐analysis ar not the original data of the literature, but the further analysis on the basis of previous research data, we cannot rule out the bias in the original research, especially the publication bias. In this study, in the meta‐analysis of neonatal respiratory diseases, both vitamin A deficiency in pathogenic literature and vitamin A supplement therapy literature found publication bias, but those two sensitivity analysis results showed that excluding any one article had no effect on the value of the pooled risk estimate, indicating that the result of meta‐analysis was stable. However, in subgroup analysis, due to the relatively small number of literatures included and some articles with large sample data, such as Chen's research [15] and Stansfied et al.'s research [25], the reliability of the study will be affected to a certain extent. But generally speaking, the results of sensitivity analysis showed that the conclusion of this meta‐analysis is stable and credible.

## 5 Conclusion

In conclusion, with the increasing population and the rising neonatal survival rate, it is of great significance to avoid neonatal serum vitamin A deficiency and appropriate vitamin supplementation to enhance neonatal immunity, reduce neonatal respiratory diseases, and then improve the quality of life of newborns.

## Author Contributions

Yuanyuan Li, Qingfeng Zhai, and Zhongliang Li designed the research. Ruoyu Zhang performed the data collection and statistical analysis. Ruoyu Zhang and Yuanyuan Li wrote the paper. All authors read and approved the final manuscript.

## Ethics Statement

This study does not involve the human subjects directly.

## Conflicts of Interest

The authors declare no conflicts of interest.

## Data Availability

The datasets generated and/or analysed during the current study are not publicly available because the data are original data but are available from the corresponding author on reasonable request.
